# The easy-to-hard training advantage with real-world medical images

**DOI:** 10.1186/s41235-018-0131-6

**Published:** 2018-10-03

**Authors:** Brett D. Roads, Buyun Xu, June K. Robinson, James W. Tanaka

**Affiliations:** 1 Department of Compute Science, University of Colorado Boulder, 1111 Engineering Drive, ECOT 717, 430 UCB, Boulder, CO 80309-0430 USA; 2 Department of Psychology, University of Victoria, P. O. Box 1700, STN CSC, Victoria, BC V8W 2Y2 Canada; 3 Department of Dermatology, Feinberg School of Medicine, Northwestern University, 645 N Michigan Ave, Suite 1050, Chicago, IL 60611 USA

**Keywords:** Visual categorization, Melanoma diagnosis, Trial scheduling, Training procedure, Difficulty prediction

## Abstract

Many medical professions require practitioners to perform visual categorizations in domains such as radiology, dermatology, and neurology. However, acquiring visual expertise is tedious and time-consuming and the perceptual strategies mediating visual categorization skills are poorly understood. In this paper, the *Ease* algorithm was developed to predict an item’s categorization difficulty (*Ease* value) based on the item’s perceptual similarity to all within-category items versus between-category items in the dataset. In this study, *Ease* values were used to construct an easy-to-hard and hard-to-easy training schedule for teaching melanoma diagnosis. Whereas previous visual training studies suggest that an easy-to-hard schedule benefits learning outcomes, no studies to date have demonstrated the easy-to-hard advantage with complex, real-world images. In our study, 237 melanoma and benign images were collected for training and testing purposes. The diagnostic accuracy of images was verified by an expert dermatologist. Based on their *Ease* values, the items were grouped into easy, medium, and hard categories, each containing an equal number of melanoma and benign lesions. During training, participants categorized images of skin lesions as either benign or melanoma and were given corrective feedback after each trial. In the easy-to-hard training condition, participants learned to categorize all the easy items first, followed by the medium items, and finally the hard items. Participants in the hard-to-easy training condition learned items in the reverse order. Post-training results showed that training in both conditions transferred to the classification of new melanoma and benign images. Participants in the easy-to-hard condition showed modest advantages both in the acquisition and retention of the melanoma diagnosis skills, but neither scheduling condition exhibited a gross advantage. The *Ease* values of the items predicted categorization accuracy after, but not before training, suggesting that the *Ease* algorithm is a promising tool for optimizing medical training in visual categorization.

**Electronic supplementary material:**

The online version of this article (10.1186/s41235-018-0131-6) contains supplementary material, which is available to authorized users.

## Significance

Numerous medical professions require practitioners to perform visual categorizations for their domain. For example, dermatologists must recognize whether a skin lesion is malignant or benign. However, the acquisition of visual expertise can be time-consuming. This work aims to develop practical training procedures that reduce the learning burden placed on medical professionals. Traditionally, it is impractical to implement a training procedure that assumes the difficulty of learning each image is known. The *Ease* algorithm provides a novel, cost-effective measure for computing image difficulty and overcoming traditional limitations. The *Ease* algorithm is a simple category learning model that predicts the probability of a participant making a correct classification. Importantly, the *Ease* algorithm incorporates both the within-category and between-category variabilities. The proposed method for computing image difficulty makes it practical to implement and compare different difficulty-based scheduling policies for real-world medical images. This work demonstrates how *Ease* values can be used to compare two commonly used schedules: an *easy-to-hard* and a *hard-to-easy* schedule. Results from a human training experiment provide no direct evidence in favor of one schedule.

## Background

It has been estimated that it takes the average person 10,000 h of training (20 h for 50 weeks a year for ten years = 10,000 h) to become an expert (Ericsson, Krampe, & Tesch-Römer, 1993). Of course, this number is not fixed. Characteristics of the learner, such as their native ability and motivation, can affect the amount of practice needed to achieve mastery. The complexity of the skill domain will also influence how much practice time is required for a person to become an expert (e.g. more practice time is needed to become a chess expert than a checkers expert). Finally, the schedule and structure of the training itself will also affect the length of training for expertise.

Previous work has demonstrated that the order of training trials influences the efficiency of the training and the trainee’s ability to visually categorize *trained* and *novel* exemplars (e.g. Birnbaum, Kornell, Bjork, & Bjork, 2013; Carvalho & Goldstone, 2014, 2015; Kang & Pashler, 2012; Pashler & Mozer, 2013; Wahlheim, Dunlosky, & Jacoby, 2011; Zulkiply & Burt, 2013). A common finding in the literature is that individuals who are trained with an easy-to-hard schedule demonstrate better transfer than groups who are trained exclusively with hard trials or with a hard-to-easy schedule. The easy-to-hard effect has been demonstrated in a variety of species, including dogs (Pavlov, 1927), pigeons (Lawrence, 1952), and rats (Liu, Mercado, Church, & Orduna, 2008). Easy-to-hard training is also more efficient if the transition from easy to difficult items is gradual rather than abrupt (Lawrence, 1952). In humans, the easy-to-hard phenomenon has been shown in visual (Hornsby & Love, 2014; McLaren & Suret, 2000) and auditory (Church, Mercado, Wisniewski, & Liu, 2013; Liu et al., 2008) modalities. To account for the easy-to-hard effect, it has been hypothesized that the easy items provide good information about category structure, are easily encoded in memory, and serve as the foundation for learning more difficult items (Avrahami et al., 1997; Hornsby & Love, 2014).

In striking contrast, other researchers have argued that a hard-to-easy schedule is more efficient for teaching complex perceptual categories (Lee et al., 1988; Spiering & Ashby, 2008). For example, when asked to classify sine-wave gratings that combined the dimensions of width and orientation, participants who began with hard items showed better learning rates and superior transfer compared to participants who were exposed to the easy-to-hard learning condition (Spiering & Ashby, 2008). It is hypothesized that the difficult items encountered early in training forced participants to focus on complex categorization rules and to quickly discard simple, one-dimensional approaches. If categories follow explicit, verbal rules, performance is equivalent regardless of whether the individuals are taught with an easy-to-hard, hard-to-easy, or random schedule of learning (Ashby, Alfonso-Reese, Turken, & Waldron, 1998; Spiering & Ashby, 2008). Hence, for the learner, the optimal schedule is determined by the structure of the to-be-learned categories.

Melanoma, a lethal skin cancer when not detected in the early curable phase, provides an ideal category domain for testing the predictions of the easy-to-hard and hard-to-easy learning methods. The conventional approach for teaching the diagnostic features of melanoma is explicitly rule-based. The ABCDE system directs observers to five key features of a lesion: its asymmetrical shape (A); irregular border (B); variegated color (C); a size that is > 6 mm in diameter (D); and evolving appearance (E). However, training programs designed to improve ABCDE diagnostic skills of physicians, medical students, and practitioners have been largely ineffective (Rourke, Oberholtzer, Chatterley & Brassard, 2015). The primary weakness of the ABCDE approach is that benign lesions may exhibit cancerous features (e.g. asymmetry, jagged borders, multi-colored, > 6 mm), and melanoma lesions, especially in their early stages of development, do not always fit the ABCDE criteria. Not surprisingly, studies have shown that even expert judges show relatively poor inter-observer reliability when evaluating a lesion for its ABCD rules of variegated color and irregular contour (Meyer, Piepkorn, Goldgar, Lewis, Cannon-Albright, et al., 1996). Rather than being a ruled-based category, melanoma and benign lesions belong to the class of “fuzzy” categories where the visual features are overlapping, probabilistic, and require perceptual integration (Ashby & O’Brian, 2005; Rosch, 1973; Rosch & Mervis, 1975; Zadeh, 1965).

One challenge with comparing different difficulty-based schedules is that it is non-trivial to determine a difficulty score for every item in a real-world image dataset. Typically, item difficulty is obtained using one of two relatively time-intensive and labor-intensive methods. One approach is to run an initial norming study with novice participants who are trained to perform the categorization task and record the accuracy statistics for each item (e.g. Lindsey, Mozer, Huggins, & Pashler, 2013). One limitation of the norming approach is that difficulty scores can only be obtained after participants are trained to a pre-specified level of performance and the difficulty scores will change if new items are added to the set. Alternatively, experts can be consulted to rank the relative difficulty of category items (e.g. Evered, Walker, Watt, & Perham, 2014). However, researchers must find qualified experts and may not have the financial resources to compensate experts for their services. In this study, an innovative alternative is introduced that avoids these drawbacks. Based on the independent judgments of naive raters, the *Ease* algorithm uses a multi-dimensional feature representation based on the image’s perceived visual similarity to all images in the dataset. The *Ease* algorithm yields scores that reflect the categorization difficulty of all images in the dataset and can be leveraged by a training schedule.

The current study compares the effectiveness of easy-to-hard and hard-to-easy training schedules in a visual category learning task using real-world skin lesion images. First, Experiment 1 describes a novel *Ease* algorithm that estimates the difficulty of an item by using a multi-dimensional feature representation that captures the visual similarity between items. In Experiment 2, the computed *Ease* values are used to construct the easy-to-hard and hard-to-easy training schedules. The two training schedules are evaluated based on their effectiveness in teaching melanoma diagnosis and retention of the diagnosis performance.

## Experiment 1: Computation of ease values

The method for predicting the difficulty of every item in a pre-defined image dataset is presented in this experiment. The difficulty of an exemplar is computed in three stages. First, a set of human similarity judgments are collected. Second, a psychological representation of similarity using human similarity judgments is inferred. Third, the inferred psychological representation of similarity is reused in a simple category learning model in order to predict the difficulty associated with each exemplar. Each of these three stages is discussed in turn and corresponding results are presented for a pigmented skin lesion image dataset. The recovered psychological representations reveal reasonable predictions that are further validated by Experiment 2.

### Methods

#### Image dataset

Images of four types of melanoma (acral lentiginous, lentigo maligna, nodular, superficial spreading) and four types of benign pigmented lesion (blue nevi, lentigo, melanocytic nevi, seborrheic keratoses) were used in the current study. Images of skin lesion were collected via Google image search by using the name of the lesion type (e.g. lentigo maligna) as the key words. The accuracy of the diagnosis of all the images were then validated by an expert dermatologist. This validation procedure excluded 11 images from the study, either because of the uncertainty of the diagnosis from visual inspection of the images alone or the existence of more than one type of melanoma lesion in the same image (e.g. lentigo maligna with a nodular component). The remaining 237 images (120 melanoma, 117 benign) were scaled to fit within a frame of 300 × 300 pixels and cropped to remove any body part information.

#### Collection of human similarity judgments

**Fig. 1:**
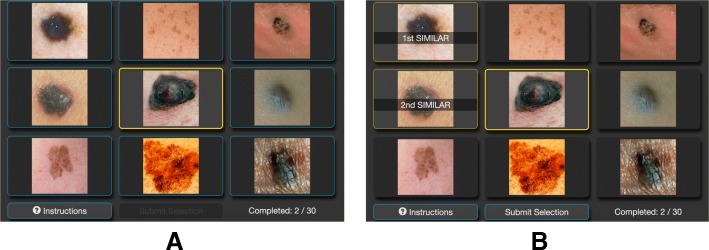
Example similarity judgment displays shown to individuals. The center image is the query image while the surrounding images are the reference images. Participants select the two reference images that are most similar to the query. **a** Initially only the query is highlighted. **b** After the participant makes their selection, the selected references are also highlighted

In the first stage, human similarity judgments are collected for the images of interest. Inspired by approaches used in the computer vision community (e.g. Wah et al., 2014), human similarity judgments are collected by having participants view displays composed of nine images arranged in a 3 × 3 grid (Fig. 1). Each display is composed of a query image (center image) and eight reference images (surrounding images). Participants are asked to select the two reference images they believe are most similar to the query image. When participants make their selection, they also indicate which reference is most similar and second most similar. Each participant evaluates multiple displays. The images for each display are selected randomly from the set of all possible images. A sufficient number of displays are evaluated such that every image occurs in at least one display.

A participant’s choices for the *i*th judged display is recorded using a vector of the following form:

$$ {\mathcal{D}}_i=\left(q,a,b,c,d,e,f,g,h\right), $$

where *q* is a scalar indicating the query image and *a*- *h* are scalars indicating the reference images. The variables *a* and *b* represent the references that the participant chose as the most similar and second most similar. The set of all judged displays, across multiple participants, is indicated by $\mathcal{D}$.

#### Inference of a psychological embedding

In the second stage, the set of all judged displays, $\mathcal{D}$, is used to infer a psychological representation of similarity, referred to as a *psychological embedding*. A psychological embedding is a multi-dimensional feature representation that models the similarity between items. The *i*th item is represented as a feature vector ***z***_*i*_, which we refer to as an embedding point. The entire embedding is denoted by the matrix ***Z***. The inference objective is to recover an embedding (***Z***) such that similar items are located closer together than dissimilar items. While there are an infinite number of potential visual features, the algorithm identifies the subset of salient features that sufficiently capture human-perceived similarity.

Many algorithms exist for determining a psychological embedding, such as metric multidimensional scaling (e.g. Glower, 1966; Torgerson, 1958), non-metric multidimensional scaling (e.g. Kruskal, 1968a, 1968b), and *t*-distributed stochastic triplet embedding (Van Der Maaten & Weinberger, 2012). Different embedding algorithms make different assumptions about the way humans perceive similarity. For example, the t-distributed stochastic triplet embedding procedure assumes that the similarity between two embedding points is described by an unnormalized Student’s t-distribution. In this work, the similarity function is constrained by existing psychological theory. Following Roads and Mozer (2016), various psychological models (e.g. Jones, Love, Maddox, 2006; Jones, Maddox, Love, 2006; Nosofsky, 1986; Shepard, 1987) are integrated into a general form to obtain:

$$ s\left({\boldsymbol{z}}_i,{\boldsymbol{z}}_j\right)=\exp \left(-\beta {\left\Vert {\boldsymbol{z}}_i-{\boldsymbol{z}}_j\right\Vert}_{\rho, \boldsymbol{w}}^{\tau}\right)+\gamma, $$

where *β*, *ρ*, *τ*, and *γ* are free parameters that control the gradient of generalization. The norm ‖***z***_*i*_ − ***z***_*j*_‖_*ρ*, ***w***_ denotes the weighted Minkowski distance where the parameter *ρ* controls the type of distance (e.g. *ρ* = 2 yields Euclidean distance). The vector ***w*** is used to model attention weights. The attention weights allow different feature dimensions to be given varying importance in determining similarity. The weight vector is constrained such that each element *w*_*i*_ ≥ 0 and ∑_*i*_*w*_*i*_ = *D*, where *D* is the dimensionality of the embedding. Points that are closer together in embedding space will have a higher similarity than points that are farther apart. For conciseness, the free parameters controlling the similarity function (i.e. *β*, *ρ*, *τ*, and *γ*) are denoted by the set variable ***θ***.

Given a psychologically motivated similarity function, it is possible to specify a simple behavioral model that predicts the choices participants make when judging displays. The likelihood of subject selections is modeled in the same spirit as Luce’s ratio of strengths formulation (Luce, 1959). For a given trial, the probability of selecting a given reference is determined by the similarity between the query stimulus and that reference. References that are more similar to the query have a higher probability of being selected. Since participants make two selections, the likelihood is a product of the probability of making the first selection and the probability of making the second selection:

$$ p\left({\mathcal{D}}_i|\boldsymbol{Z},\boldsymbol{\theta} \right)=\frac{s\left({\boldsymbol{z}}_q,{\boldsymbol{z}}_a|\boldsymbol{\theta} \right)}{\sum_{r\in {\mathcal{R}}_i}s\left({\boldsymbol{z}}_q,{\boldsymbol{z}}_r|\boldsymbol{\theta} \right)}\frac{s\left({\boldsymbol{z}}_q,{\boldsymbol{z}}_b|\boldsymbol{\theta} \right)}{\sum_{r\in {\mathcal{R}}_i\neg a}s\left({\boldsymbol{z}}_q,{\boldsymbol{z}}_r|\boldsymbol{\theta} \right)}. $$

The set variable ${\mathcal{R}}_i$ indicates the set of all references *a*–*h* that were presented on the *i*th display. Before making the first selection, participants have eight choices. After making the first selection, participants must choose from among the seven remaining references (${\mathcal{R}}_i\neg a$). The likelihood of all the judged displays is given by:

$$ p\left(\left.\mathcal{D}\right|\boldsymbol{Z},\boldsymbol{\theta} \right)=\prod \limits_{\boldsymbol{i}}p\left(\left.{\mathcal{D}}_i\right|\boldsymbol{Z},\boldsymbol{\theta} \right). $$

To infer a psychological embedding, gradient decent is used to find the set of parameters ***Z*** and ***θ***, that maximizes the log-likelihood:

$$ \underset{\boldsymbol{Z},\boldsymbol{\theta}}{\max}\sum \limits_i\log p\left(\left.{\mathcal{D}}_i\right|\boldsymbol{Z},\boldsymbol{\theta} \right). $$

By maximizing the log-likelihood the algorithm produces a set of embedding points and a corresponding similarity function that emulates human-perceived similarity. One drawback of many embedding algorithms is that the dimensionality must be specified beforehand. The embedding algorithm presented here is no exception. In order to determine the dimensionality of the embedding, a separate embedding is inferred using different dimensionality settings. Each embedding is tested on its ability to predict a held-out set of similarity judgments using a threefold cross-validation procedure. The dimensionality that results in the best predictions is selected.

During inference, the attention weights provide an unnecessary degree of freedom. The embedding algorithm is capable of stretching and contracting the space without the attention weights. If the similarity judgments were derived from distinct populations (e.g. novices and experts), a unique set of weights could be inferred for each population, in the same spirit as the INDSCAL algorithm (Carroll & Chang, 1970). Since it is assumed that there is only one population, all attention weights are fixed to one. The attention weights are included in the formulation of similarity because they play an important role in the next stage.

#### Prediction of item difficulty

In the third stage, a simple category learning model is used to predict the difficulty of learning each item in the image dataset. In principle, any category learning model that predicts the probability of a correct categorization can be used to predict item difficulty (e.g. Love, Medin, & Gureckis, 2004; Nosofsky, 1986, Shepard, 1987). The probability of correct categorization can be used as a direct measure of difficulty. If a category learning model predicts a low probability of correct categorization, then the item is relatively difficult. Conversely, if the category learning model predicts a high probability of correct categorization, then the item is relatively easy. However, category learning models have free parameters that must be fit using behavioral data. In contrast to typical approaches, the free parameters of the proposed model are determined by a previously inferred psychological embedding.

The proposed category learning model is a simplified version of the Generalized Context Model (Nosofsky, 1986). Since the free parameters of the proposed model are not being fit in the typical manner, we refer to the model as the *Ease* algorithm. The *Ease* algorithm leverages similarity functions to predict categorization probabilities and therefore a measure of difficulty. The *Ease* algorithm assumes that every stimulus has an embedding point ***z***_*i*_ and a corresponding label *y*_*i*_ that indicates its category membership. The *Ease* algorithm predicts the probability that the *i*th stimulus will be categorized correctly:

$$ {e}_i=\frac{\sum_{j\in {\mathcal{S}}_i}s\left({\boldsymbol{z}}_i,{\boldsymbol{z}}_j\right)}{\sum_{k\in {\mathcal{I}}_{\neg i}}s\left({\boldsymbol{z}}_i,{\boldsymbol{z}}_k\right)}, $$

where ${\mathcal{S}}_i=\left\{l\in {\mathcal{I}}_{\neg i}|{y}_l={y}_i\right\}$ is the set of indices that belong to the *same* category as the *i*th image and $\mathcal{I}$ is the set of indices representing all the images in the embedding. The numerator of the Ease algorithm adds up the similarity between the *i*th stimulus and all other stimuli that belong to the same category $\Big({\mathcal{S}}_i$), effectively producing a measure of within-category similarity. The denominator of the Ease algorithm adds up the similarity between the *i*th stimulus and all other stimuli (${\mathcal{I}}_{\neg i}$). The Ease algorithm therefore compares how similar a stimulus is to its own category members relative to all possible categories. Importantly, the Ease algorithm uses the same similarity function as the embedding procedure.

The same similarity function is employed in the *Ease* algorithm and the choice model of the embedding procedure. This aspect enables the reuse of the similarity function learned during the embedding procedure, with one consequential change. The *Ease* algorithm uses different attention weights. Following the approach used by Nosofsky (1986), it is possible to compute the optimal attention weights for a rational agent performing a categorization task. The rational weights can be viewed as the attention weights that an expert would use during categorization. The reused similarity function, embedding points, and rationally optimal weights produce a simple model capable of generating *Ease* values.

Reusing a similarity function fit by similarity judgments provides an advantage because similarity judgments are easier to collect and reusable if the set of images changes. The protocols for collecting similarity judgments can be much shorter than training protocols. A training protocol must be sufficiently long to observe a change in performance. This means that participants must complete an entire (typically lengthy) training protocol in order to be included in the fitting procedure. In contrast, the protocol for collecting similarity judgments can be arbitrarily short. Shorter protocols mean a smaller time commitment for participants, potentially increasing the pool of willing participants.

A second advantage concerns reusability. In general, the difficulty of an exemplar depends on the other exemplars used in the experiment. Adding exemplars can change the location of category boundaries and the degree of overlap between different categories. If a researcher derives difficulty scores from training data, but later decides to use an expanded set of stimuli, the previously derived difficulty scores may no longer reflect the actual difficulty of the task. In contrast, all previously collected similarity judgments can be reused. To infer an updated embedding, more similarity judgments need to be collected that include the new stimuli. Then, a new embedding is inferred using the expanded set of similarity judgments. Inferring a new embedding is computationally inexpensive (about 5 min for the dataset used in Experiment 1). Lastly, a psychological embedding is extremely versatile. While this work focuses on using the inferred embedding in order to make difficulty predictions, the embedding itself can also be used in more sophisticated cognitive modeling and analysis of visual features.

### Results and discussion

**Fig. 2:**
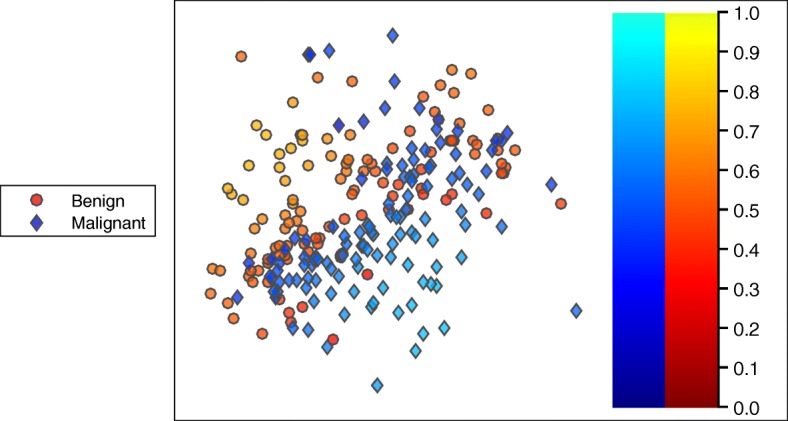
An inferred two-dimensional embedding for 237 skin lesion images. Each *dot* represents a unique image where the color of the dot indicates the image category (*reddish*: benign, *bluish*: malignant). The brightness of the color indicates the *Ease* value of the exemplar. The brighter colors (*yellow* and *cyan*) indicate exemplars that are relatively easy. The darker colors indicate exemplars that are relatively difficult

Using the previously described approach, an inferred psychological embedding was obtained for an image dataset composed of 237 skin lesion images (120 melanoma, 117 benign). Similarity judgments were collected from 112 novice participants on Amazon Mechanical Turk. Each participant completed 27 trials, yielding 3024 judged displays. The sample size of 112 participants was chosen based on computer simulations using a known, synthetic ground truth. Applying the embedding algorithm to the collected similarity judgments yields a three-dimensional psychological embedding. A two-dimensional visualization of the embedding (Fig. 2) illustrates the high degree of visual feature overlap between benign and malignant images, indicative of a difficult category learning task.

**Fig. 3:**
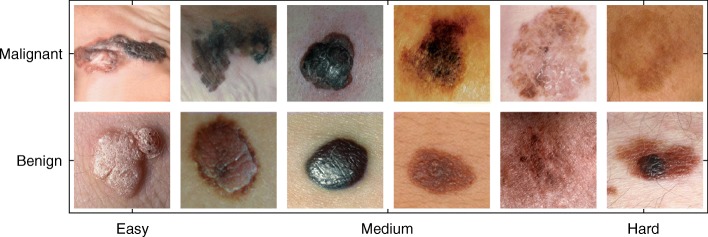
A set of images demonstrating the spectrum of difficulty for the two classes of skin lesions. The *first* and *second row* show malignant and benign skin lesions, respectively. Within each row, the items on the *left* are predicted to be the easiest while the items on the *right* are predicted to be the hardest

Once the psychological embedding has been obtained, the inferred similarity function and embedding points are reused in the *Ease* algorithm. The *Ease* algorithm predicts the relative difficulty of each image in the image dataset. The items predicted to be the easiest are surrounded by neighbors of the same class (Fig. 2). Items that are predicted to be difficult have neighbors from the opposite class. The overlap of visual features can be partially understood by visually examining images with a spectrum of predicted *Ease* values (Fig. 3). The hardest items from both categories tend to exhibit visual features that are common to both categories.

## Experiment 2: Comparing easy-to-hard and hard-to-easy training schedules

Visual learning is a critical skill in medical diagnosis education. For example, neurologists make diagnostic decisions by viewing magnetic resonance scans, radiologists analyze mammograms for evidence of cancer and dermatologists inspect skin lesions for melanoma. Anecdotally, medical educators often introduce more typical cases first—those that have the classic representation of the symptom—before introducing more atypical cases. This implies that an easy-to-hard schedule might already be in use by medical educators. One medical training study in cytopathology (Evered et al., 2014), manipulated the difficulty of the training items and suggested that training should avoid images along category boundaries. However, it was unclear whether the easy-to-hard schedule was superior to the hard-to-easy schedule.

The purpose of the present training task was to teach trainees to make correct diagnosis of whether a pigmented lesion is melanoma or benign. Previously Xu, Rourke, Robinson, and Tanaka (2016) have shown that trainees can improve significantly in melanoma diagnosis after receiving perceptual training with the exposure to multiple exemplars of pigmented skin lesion images, with immediate feedback of the correctness of the diagnosis, and with the requirement to reach the accuracy criterion of 90% with all the training images. In this study, instead of scheduling training items randomly, training items are introduced following either the easy-to-hard or the hard-to-easy schedule.

Experiment 2 directly compares the performance and knowledge retention of groups trained using an easy-to-hard training schedule and a hard-to-easy training schedule. Categorizing lesions is a good test of the predictions of two types of scheduling procedures because it requires integration of information across multiple dimension (e.g. size, coloration, symmetry, and contour) (Ashby & Spiering, 2004; Spiering & Ashby, 2008). When categorization requires perceptual integration, some studies found a learning advantage for the easy-to-hard approach (Church et al., 2013; Liu et al., 2008; McLaren & Suret, 2000), while others report a learning advantage for the hard-to-easy approach (Spiering & Ashby, 2008). In the current experiment, all participants received the same number of easy, medium, and difficult training trials. Participants in the easy-to-hard group were trained with the easy items first, followed by the medium items and hard items. Participants in the hard-to-easy group learned items in the reverse order. Item difficulty was determined using the *Ease* values of the skin lesion images obtained in Experiment 1. Pre-training and post-training performance for the two schedules was tested immediately after training and two weeks later. The pre-training and post-training performance was correlated with the *Ease* value of individual test items. The two training conditions were compared by examining overall performance, as well as difficulty-specific performance, in the immediate and two-week post-test.

### Method

#### Participants

Based on an a priori power analysis using the criteria of Cohen’s d = 0.8 (large effect size, Cohen, 1988), alpha = 0.05, power = 0.8, and an attrition rate of 20%, we planned to test 31 participants in each of the training conditions. Sixty-two undergraduate students from the University of Victoria participated in the study. All of the participants had normal or corrected-to-normal vision and none of them have received formal medical training. Thirty-one participants (seven men) were randomly selected to participate in the easy-to-hard condition and another 31 participants (10 men) participated in the hard-to-easy condition. The average age of the easy-to-hard (*M* = 22.7, *SD* = 5.4) and hard-to-easy (*M* = 21.8, *SD* = 4.4) was not significantly different (*t*_60_ = 0.72, *p* = 0.47, Cohen’s *d* = 0.18), nor were the gender ratios significantly different (*χ*^2^ = 0.73, *p* = 0.40).

#### Melanoma diagnosis test (MDT)

The MDT is a measure of the ability to discriminate between melanoma and benign pigmented skin lesion images. In the MDT, six images of each of the four types of melanoma and benign lesions were selected (48 images in total) from the image pool. A mixture of easy, medium, and hard items was selected for melanoma and benign lesions. The melanoma and benign lesions had an average *Ease* value of 0.60 (*SD* = 0.10) and 0.57 (*SD* = 0.12), respectively. In each trial, participants saw one skin lesion image and were asked to judge whether the lesion was “Benign” or “Melanoma” by clicking the buttons presented under the image. The MDT served as the pretest (before training), immediate post-test (immediately after training), and delayed post-test (two weeks after training). The images were identical in all three tests, with the exception that images were rotated 90° clockwise for the immediate post-test and 180° clockwise for the two-week post-test. Images used in the MDT were never used in the training.

#### Training

Twelve images of each of the four types of melanoma and benign skin lesions were used for training (96 in total). Images used during training were never used in the MDT. All the benign and melanoma lesion images were first sorted by their *Ease* values. Sixteen of the melanoma images (regardless of their sub-types) with the highest *Ease* value were labeled as easy items, 16 of the melanoma images with the lowest *Ease* value were labeled as hard items, and the remaining 16 melanoma images were labeled as medium items. The same method was used to group the benign lesions. As a result, for melanoma lesions, the easy, medium, and hard items had *Ease* values of 0.72 (*SD* = 0.06), 0.59 (*SD* = 0.03), and 0.49 (*SD* = 0.05), respectively. For benign lesions, the easy, medium, and hard items had *Ease* values of 0.72 (*SD* = 0.08), 0.56 (*SD* = 0.03), and 0.43 (*SD* = 0.07), respectively. Each of the easy, medium, and hard training blocks contains 16 melanoma and 16 benign lesion images. In the training, participants in the easy-to-hard (hard-to-easy) condition received four iterations of the easy (hard) training block, followed by four iterations of the medium training block, and, finally, four iterations of the hard (easy) training blocks. When a training block was repeated, the same images were used as in the previous block, but appeared randomly in one of the four orientations (i.e. upright, inverted, rotated 90° clockwise, and rotated 90° counterclockwise). As a result, all participants had 384 trials during training. In each trial, participants were required to decide whether the lesion image presented on the screen was melanoma or benign by clicking on the “Melanoma” or “Benign” buttons presented underneath the image. Feedback about the accuracy of the diagnosis was provided immediately after participants responded.

#### Procedure

**Fig. 4:**
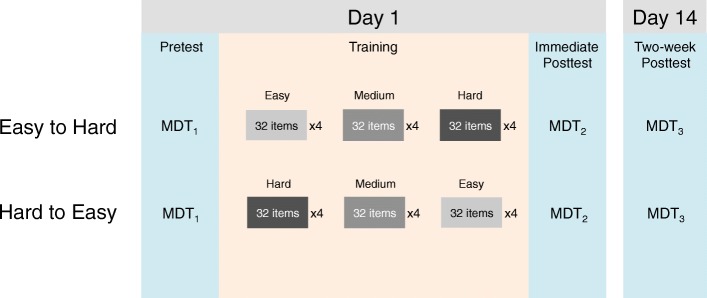
An *illustration* of the training and testing schedule used in Experiment 2. *Blue boxes* represent the MDT. *Gray boxes* represent the block of training items used in each training session. The lighter the color, the easier the items

The detailed training and pre/post-test arrangements were illustrated in Fig. 4. All 62 participants visited the lab on day 1. They first took the MDT as the pretest. Then, they were randomly assigned into the easy-to-hard and hard-to-easy conditions for the training. After they completed the training, they were given the MDT as the immediate post-test. All participants were invited to complete the second post-test 14 days after the first post-test remotely using their own computers. Both the MDT and the training were programmed using the JsPsyche library (de Leeuw, 2015) using JavaScript and deployed using an online data collection platform developed by the lab led by the senior author of this study. Skin lesion images were 300 × 300 pixels in size. On day 1, the pretest, training, and immediate post-test were conducted in the lab. Participants viewed the images on a 22-inch monitor with a resolution of 1680 × 1050 pixels at a viewing distance of approximately 70 cm, resulting in a visual angle of 6.9° × 7.0°. However, no specific instruction was given to require the participants to remain at this viewing distance during the experiment. The two-week post-test was done remotely so the size of display and viewing distance were unknown.

### Results and discussion

#### Improvement across training sessions

**Fig. 5:**
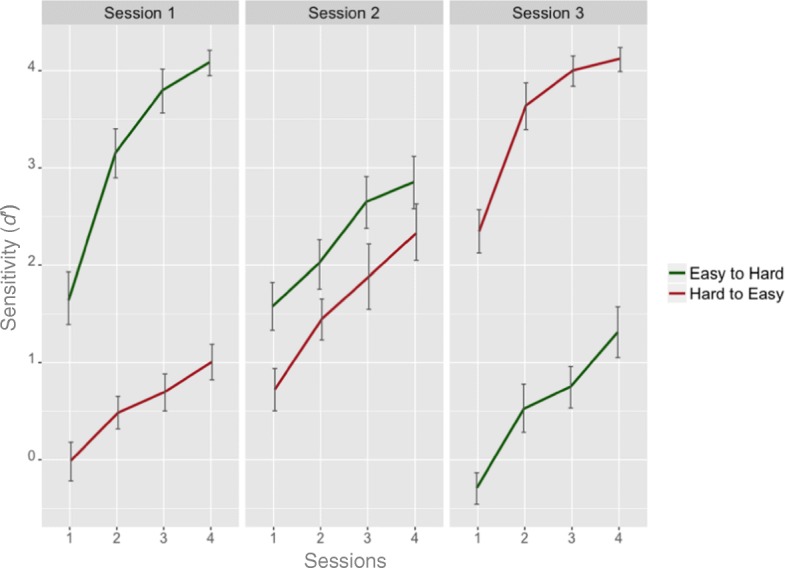
The performance (as measured by d’) throughout the training for the easy-to-hard and hard-to-easy training group. *Error bars* denote 95% confidence intervals

Using correct detection of melanoma as a hit (H) and categorizing benign lesion as melanoma as false alarm (FA), sensitivity (*d′*) could be calculated for each individual as the difference between the Z transforms of the H rate and the Z transforms of the FA rate (i.e. *d*′ = *Z*_H_ – *Z*_FA_). The measure of d’ was calculated for each training block across all three training sessions (Fig. 5). Visual inspection suggested that in each session, training performance improved continuously in both groups. Moreover, in Session 1 where the easy-to-hard (hard-to-easy) group repeated four blocks of training with easy (hard) items, training performance was better in the easy-to-hard than the hard-to-easy group. This pattern was mirrored in Session 3 where the easy-to-hard (hard-to-easy) group repeated four blocks of training with hard (easy) items and training performance was better in the hard-to-easy than the easy-to-hard group. Interestingly, in Session 2 where both groups of participants repeated four blocks of items of medium level difficulty, the easy-to-hard group performed better than the hard-to-easy group. This observation was further investigated by a 3 × 2 ANOVA and examining the two-way interactions between the within-subject variable of Sessions (Session 1–3) and the between-subject variable of Training Policy (easy-to-hard versus hard-to-easy). This interaction was significant (*F*_(2,120)_ = 854.51, *p* < 0.001, *η*^2^= 0.85). Multiple Bonferroni corrected comparisons were conducted between the two groups for each session. There were significant group differences in all sessions: Session 1 (*t*_*60*_ = 23.93, *p* < 0.001, Cohen’s *d* = 6.07); Session 2 (*t*_*60*_ = 4.51, *p* < 0.001, Cohen’s *d* = 1.15); and Session 3 (*t*_*60*_ = − 26.35, *p* < 0.001, Cohen’s *d* = 6.69).

The most important finding from the training data was from the between-group comparison in Session 2, which provides evidence regarding the advantages of the easy-to-hard policy. Unlike Session 1 or Session 3, both groups were trained using the exact same items in Session 2. Before Session 2, the two groups of participants had different training experience. In Session 1, the easy-to-hard group was trained with the easy items whereas the hard-to-easy group was trained using the hard items. Therefore, any differences emerging from the between-group comparison can only be attributed to the different training history for the two groups. These results suggest that learning the easy items first established a better foundation for the trainees to learn the medium difficulty items in the subsequent session.

#### Post-training gain

**Fig. 6:**
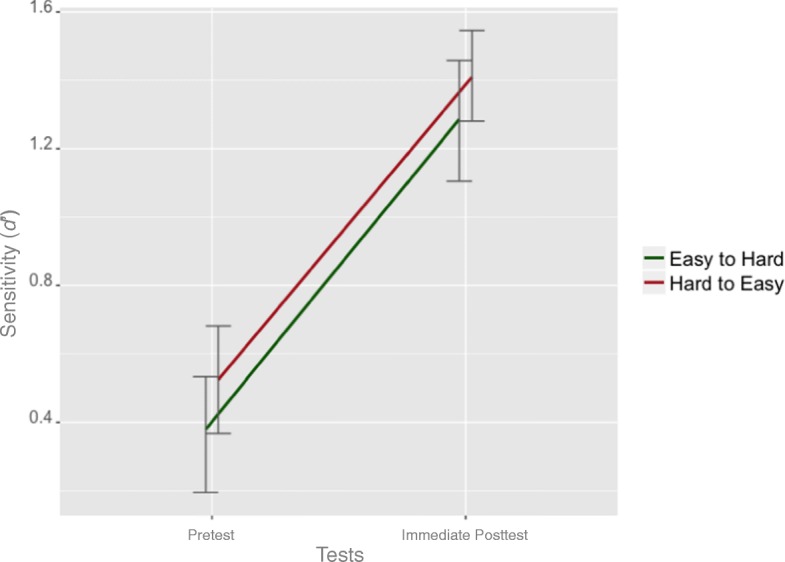
The sensitivity (d’) in the pretest and post-test of MDT for participants receiving the easy-to-hard and hard-to-easy training. *Error bars* stand for 95% confidence intervals

The sensitivity (d’) measure was used to compare the performance in MDTs administered before and immediately after the training. A 2 × 2 ANOVA was conducted, with Test (pre versus post) as within-subject variable and Training Policy (easy-to-hard versus hard-to-easy) as between-subject variable (Fig. 6). The main effect of Test (*F*_(1,60)_ = 145.32, *p* < 0.001, *η*^2^= 0.49) was significant, indicating both groups improved after the training. However, neither the main effect of Training Policy nor the interaction between Test and Training Policy were significant (all *F*s < 2.1, all *p*s > 0.15). Similar results were found when H and FA rates were analyzed separately, with only the main effect of Test being significant. The results from the direct comparison between the performance in the pretest and post-test show that both the easy-to-hard and hard-to-easy training policy were able to improve overall melanoma diagnosis performance to the same degree.

#### *Ease* value predictions

Another important question is whether the *Ease* values can accurately predict diagnosis performance of the lesion images. If the *Ease* values are a good predictor of the diagnosis difficulty of the lesion images, participant performance should correlate with the predictions. It was also hypothesized that the MDT items’ accuracy should not correlate significantly with the *Ease* value in the pretest MDT, but the correlation should be significantly larger with same items in the post MDTs. The *Ease* algorithm effectively constitutes a simple model of an expert. Before training, participant performance should correlate poorly with the predictions of an expert model. In contrast, trained participant performance should correlate highly with the predictions of an expert model.

**Fig. 7:**
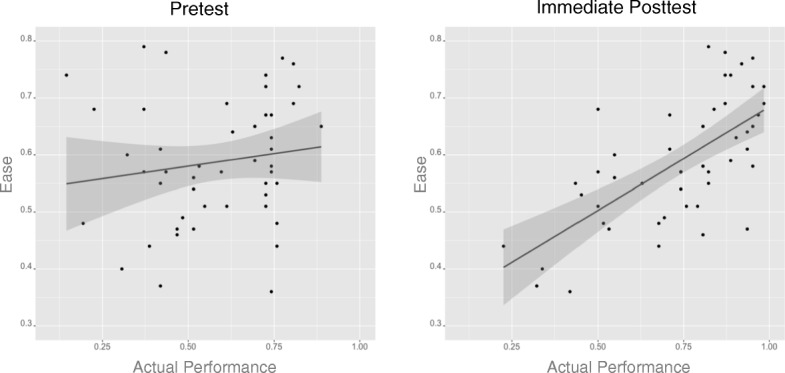
The *scatter plots* depicting the correlation between the *Ease* value and the actual performance in pretest and immediate post-test of each of the 48 items used in MDT

The *Ease* values of each of the 48 items in the MDT were used to correlate with the actual performance on those items in the pretest, immediate post-test, and two-week post-test. All 62 participants’ data were used to compare the correlations between the *Ease* values and performance in the pretest and *Ease* values and performance in the immediate post-test (Fig. 7). The results showed that the *Ease* value did not significantly correlate between the accuracy of the items in pretest MDT (*r* = 0.15, *p* = 0.32), but correlated significantly with accuracy of the items in immediate post-test (*r* = 0.66, *p* < 0.001). The difference between these two correlation coefficients was significant (*p* < 0.005), indicating that the improved correlation is due to training.

The significant correlation between *Ease* and performance in immediate post-test was further investigated between the easy-to-hard and hard-to-easy conditions. The results showed that this correlation was significant in both the easy-to-hard (*r* = 0.60, *p* < 0.001) and hard-to-easy conditions (*r* = 0.68, *p* < 0.001), but the correlation coefficients of the two groups were not significantly different (*p* = 0.52). For both training policies, equivalent and significant correlations were found between the Ease values and actual performance in the post-test MDT, but not in the pretest MDT. This suggests that a participant’s internal representation of the category structure became more expert-like, as measured by the predictions of the *Ease* algorithm.

#### Retention

**Fig. 8:**
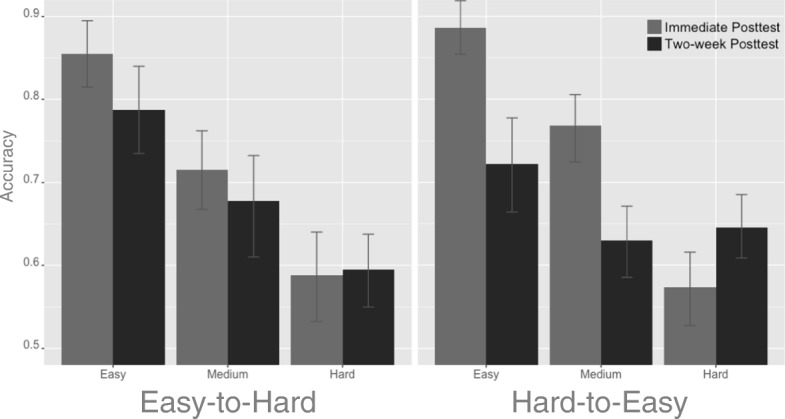
The performance change in accuracy between immediate post-test and two-week post-test in easy, medium, and hard MDT items for participants receiving the easy-to-hard and hard-to-easy training. *Error bars* stand for 95% confidence intervals

Retention was measured using data from the first post-test (immediately after training) and the second post-test (14 days after the pretest). Fifty-two (25 in easy-to-hard condition) out of 62 participants completed the two-week post-test, resulting in an attrition rate of 16%. In order to investigate the performance change between the immediate post-test and the two-week post-test in easy, medium, and hard items in the MDT separately, items in the MDT were binned into easy (16 items), medium (16 items), and hard (16 items) sets based on their *Ease* values. A 3 × 2 × 2 ANOVA was conducted with Difficulty (easy, medium, and hard) and Test (immediate post-test versus two-week post-test) as within-subject variables and Training Policy (easy-to-hard and hard-to-easy) as the between-subject variable (Fig. 8). A main effect was found for Test (*F*_(1,50)_ = 20.34, *p* < 0.001, *η*^2^= 0.05). However, the main effect of Training Policy and the two-way interactions involving Training Policy were not significant (all *F*s < 3.3, all *p*s > 0.07, all *η*^2^< 0.008), indicating that the performance for both groups dropped between the first to second post-test. Importantly, the three-way interaction between Training Policy, Test, and Difficulty was significant (*F*_(2,100)_ = 5.96, *p* < 0.01, *η*^2^=0.02). In order to further investigate this three-way interaction, retention scores were calculated as the difference between the performance at immediate post-test and two-week post-test. Multiple Bonferroni corrected t-tests on the degree of decay between the two groups showed that the easy-to-hard group had less decay in both the easy (*t*_49_ *=* 2.21, *p* < 0.05, Cohen’s *d* = 0.62) and medium items (*t*_49_ *=* 2.75, *p* < 0.01, Cohen’s *d* = 0.77) but had equivalent decay in the hard items (*t*_49_ *=* − 1.65, *p* = 0.11, Cohen’s *d* = 0.46).

In summary, both groups show equivalent overall performance drops at the two-week post-test. These results indicate that visual categorization knowledge deteriorated for participants in both groups. However, between-group differences were found when the performance decay was examined at the level of item difficulty. The easy-to-hard condition resulted in a larger amount of retained performance in easy and medium items.

## General discussion

In this study, an innovative algorithm was used to estimate the diagnostic difficulty or *Ease* value of skin lesion images. Using the *Ease* values, easy, medium, and hard items were identified and two training schedules were implemented to train the correct diagnosis of melanoma and benign skin lesions (i.e. easy-to-hard and hard-to-easy). As assessed by performance on the MDT, both training schedules were effective in reliably improving melanoma diagnosis of the trainees. Moreover, training sensitized participants to the underlying category structure differentiating melanoma and benign pigmented images and the types of lesions that are easy and difficult to classify.

For assessing item categorization difficulty, the *Ease* method has several advantages over previous norming and expertise approaches. First, whereas the source of category decisions posited by novices and difficulty ratings made by experts is uncertain, *Ease* values are explicitly derived from a representation of perceptual similarity. Item difficulty is determined relative to how perceptually *similar* an item is to other items belonging to the same melanoma (or benign) category and how perceptually *dissimilar* an item is to other items belonging to the contrasting benign (or melanoma) category. Second, whereas norming and expertise methods are relatively costly in terms of time and money, *Ease* values can be recalculated quickly allowing for new items to be readily incorporated into the dataset.

Using a sample domain of skin lesion images, it has been shown that the computed *Ease* values are predictive of the difficulty of the items both during the training and at post-test. The first source of evidence comes from the fact that participants performed significantly better with easier items during training. This result indicates that the items predicted to be difficult were actually harder to learn. The second source of evidence comes from the post-test results. After training, the specific item’s *Ease* value is predictive of its averaged accuracy across all participants, regardless of schedule condition. This suggests that the *Ease* values are capturing an aspect of difficulty that is invariant to the training schedule.

Although there were no overall post-test differences between participants in the easy-to-hard and hard-to-easy conditions, there were some intriguing differences revealed by additional analyses. First, the easy-to-hard group performed better than the hard-to-easy group in the second session of the training where the same medium items were used. One possible explanation for this difference could be that participants in the hard-to-easy group were less motivated to learn since the first training session was so difficult. Studies using the *errorless learning* approach suggest that the initial stage of the training should be easy in order to boost the learners’ confidence (e.g. Ahissar & Hochstein, 1997; Baddeley, 1992; Terrace, 1964). Although motivation was not directly measured in this study, the results show that participants in the hard-to-easy group had a steady growth in performance across the four hard blocks of training in the first training session. A steady growth in performance suggests that participants were not becoming discouraged. Moreover, participants were repeatedly informed of their progress at the end of each training block. This kind of feedback probably provides participants a better sense of their own gains, even when making many errors.

Alternatively, the difference in Session 2 training performance could be attributed to sequence-sensitive learning mechanisms. In such a scenario, the order in which items are experienced influences the structure of the current knowledge representation (e.g. Love et al., 2004). Other empirical work strongly suggests that sequence-sensitive learning mechanisms exist (e.g. Carvalho & Goldstone, 2014; Carvalho & Goldstone, 2015). Given the existence of sequence-sensitive learning mechanisms, it is still unclear how best to exploit them to promote more efficient learning. The Session 2 performance difference provides modest evidence that experience with easy items provides better scaffolding for the learning of new items.

Consistent with this interpretation, Hornsby and Love (2014) found that participants who were trained with only prototypical (i.e. easy) mammograms showed better transfer to novel easy and medium mammograms than participants who were trained with randomly presented easy, medium, and hard mammograms. Similar to our results, they found that neither prototype training nor random training transferred to the categorization of hard items (Hornsby & Love, 2014), presumably because hard items constitute exceptional cases that are perceptually dissimilar to the category prototype.

Another intriguing difference is that participants in the easy-to-hard condition appeared to retain more knowledge of easy and medium difficulty items at the second post-test. One possible interpretation of these results is that participants trained with the easy-to-hard condition preserve the prototypical category structure of their mental representations better than participants in the hard-to-easy condition. However, the current study does not provide enough evidence to resolve this conjecture. Future work is necessary to conclusively determine which scheduling policy is best for training pigmented melanoma diagnosis.

## Conclusions

By using a novel and cost-effective method to compute item difficulty, it was possible to compare an easy-to-hard and hard-to-easy training schedule with real-world images. The results showed that both training schedules were equally effective at improving the trainee’s performance in melanoma diagnosis. By using novel items during the post-tests, the results indicate that the participants acquired generalizable knowledge.

The current study makes two domain-general contributions. First, this work introduced a cost-effective procedure for predicting the difficulty associated with learning real-world medical images. Second, the current study provided an example of how difficulty predictions can be used to systematically sequence trials and potentially improve the efficiency of visual category training. Although melanoma diagnosis was used as an example case, the methods presented in this work generalize to other medical domains, such as radiology, retinopathy, electrocardiogram, and cytology. Like melanoma diagnosis, other medical domains exhibit categories that have fuzzy category boundaries. The results of the current study demonstrate that it is still possible to predict and more efficiently train domains that exhibit highly overlapping categories. The *Ease* algorithm combined with the difficulty-based schedule has the potential to reduce the costs of training personnel to make medically relevant visual categorizations.

## Additional file

Additional file 1: Supplementary material experiment_2_results. (XLSX 1040 kb)

## Abbreviations

MDT: Melanoma diagnosis test
